# A Preliminary Study for Ultraviolet Optical Methyl Salicylate Monitoring in Agriculture

**DOI:** 10.3390/s25103021

**Published:** 2025-05-10

**Authors:** Ivana Di Bari, Domenico Longo, Giuseppe D’Arrigo, Gaetano Giudice, Antonella Sciuto

**Affiliations:** 1 Consiglio Nazionale delle Ricerche, Istituto per la Microelettronica e Microsistemi (CNR-IMM), VIII Strada n. 5, Zona Industriale, 95121 Catania, Italy; giuseppealessiomaria.darrigo@cnr.it; 2 Department of Agriculture, Food and Environment of University of Catania (Di3A-UniCT), Via S. Sofia, 100, 95123 Catania, Italy; domenico.longo@unict.it; 3 Istituto Nazionale di Geofisica e Vulcanologia, Sezione di Catania, Osservatorio Etneo (INGV-OE), Piazza Roma 2, 95125 Catania, Italy; gaetano.giudice@ingv.it; 4 Centro Siciliano di Fisica Nucleare e Struttura della Materia, Via S. Sofia 64, 95123 Catania, Italy

**Keywords:** Methyl Salicylate, VOC sensing, agriculture, UV optical sensor, solid-state spectroscopy

## Abstract

Methyl Salicylate, commonly known as wintergreen oil, is a volatile organic compound which plays a crucial role in agriculture as a signaling compound for plant defense mechanisms and as an attractant for beneficial insects. Rapid and accurate detection of Methyl Salicylate is pivotal for understanding plant responses to stress and plant-to-plant communication, and it is crucial for pest management. In this work, we propose a proof of concept for Methyl Salicylate detection in agriculture, exploiting a solid-state sensor technology. Our attention is focused on the monitoring of the second characteristic Methyl Salicylate optical absorption peak, at about 310 nm. To achieve this, we developed an optical sensing apparatus comprising a UV source, a silicon carbide visible-blind UV detector, and a chamber with a 16 cm optical path. This configuration enables testing of various Methyl Salicylate concentrations and achieves a detection limit as low as 70 ppb at room temperature. Preliminary cross-testing with Methyl Jasmonate demonstrated selectivity for Methyl Salicylate, confirming the sensor’s specificity. Additionally, a design for a compact and handheld system for on-site volatile organic compound monitoring in the agricultural field is also proposed.

## 1. Introduction

Methyl Salicylate (MeSA) is produced by plants as a response to various biotic [1,2] and abiotic [3,4] stressors, including herbivory, pathogen attack and water stress, as well as some other volatile organic compounds (VOCs) [5]. Its role as a signaling molecule in plant defense mechanisms and its importance in Systemic Acquired Resistance (SAR) has been extensively studied, highlighting its significance in orchestrating plant responses to stressors [6]. Different studies about MeSA release by plants, due to pathogen attack, were performed, demonstrating that its concentration quickly increases after infection. Shulaev et al. [7] demonstrated that MeSA is the major volatile compound produced by tobacco plants inoculated with Tobacco Mosaic Virus (TMV), reaching values of tens of ppb, and Deng et al. [8,9] interestingly observed that a similar response to TMV exists in tomato plants. Moreover, MeSA serves as a pheromone, attracting beneficial insects such as parasitic wasps and predators, thereby aiding in pest control and enhancing ecological sustainability in agricultural systems [10]. The accurate and real-time detection of MeSA, as well as throughout time, is essential for several agricultural applications, including pest monitoring, disease management, and understanding plant–insect interactions [11,12]. Conventional chromatographic separation methods such as Gas Chromatography (GC) [13,14,15,16] or High-Performance Liquid Chromatography (HPLC) [17,18] coupled with MS (Mass Spectrometry) [8,9,13,14,15,16] UV-Vis [17,18] or Raman Spectroscopy [16,19] for VOC in general and, particularly, for MeSA detection, are extensively used. All these methodologies offer high sensitivity and selectivity, but they are often labor-intensive, time-consuming and require specialized equipment and trained personnel, as well as some other techniques used to preconcentrate the sample, like Solid-Phase Microextraction (SPME) [8,9,13,20,21] and thermal desorption [14,15]. All these approaches are not suitable for real-time monitoring of in-field conditions.

In the last decade, there has been growing interest in developing sensing technologies that are cost-effective, portable, and suitable for real-time monitoring in agricultural settings. Several interdisciplinary efforts have been made in this direction; in fact, different kinds of sensors were designed and made, exploiting chemical/biochemical receptors based on polymers, nanostructures, enzymes, combined with micro- and nanotechnologies [11].

Various bio-chemical sensors based on electrochemical principles are reported. Fang et al. [22,23,24] performed different studies about MeSA detection through the exploitation of enzyme-catalyzed cascade reactions through the functionalization of a Glassy Carbon Electrode (GCE) with Multiwalled Carbon Nanotubes (MWCNTs), changing, at any time, a properly chosen enzyme, and following an amperometric signal. An example of Ciclovoltammetric detection has been carried out by functionalizing a carbon screen printed electrode with 20 nm sized Au nanoparticles (NPs) [25], while Chronopotentiometric sensing systems have been investigated by Kanabasapathy et al. [26,27], firstly for pharmaceutical applications, by using a graphite pencil electrode, and a second time, by trading on the electrodeposition of Co_3_O_4_ nano-spinel films.

Different approaches have been accomplished using a Quartz Crystal Microbalance (QCM) covered with maltodextrin for a mass sensitive detection [28] or functionalizing a Molecular Imprinted Polymer (MIP) with graphene to obtain a microwave antenna sensor [29]. Another class of electrical sensors are resistive sensor arrays based on SnO_2_ thick film metal oxide, acting as receptor materials, and used to monitor VOC [30,31,32]. More recently, Jung et Park [33] presented a resistor system based on a reduced graphene oxide/poly (diallyldimethyl ammonium chloride) (rGO-PDADMAC) complex for MeSA detection. A capacitive sensor or inductor capacitor resistor (LCR) [34], based on different polymers eventually caped with Au NPs for the LCR system [35], have also been exploited.

New examples of performant sensors based on nanomaterials are reported by Zhu et al. [36] and Yang et al. [37], where the first sensing system consists of Pd-doped SnO_2_ and the second one is based on Au or Pt nanofibers covered with WO_3_, which are just a few of the cases where the MeSA concentrations tested are of 160 and 100 ppb (which are in a range close to the value typically released by plants [7,8,9]), but with operative temperatures of 250 and 450 °C, respectively.

The last class of sensors is optical-based. Different nanosized systems which exploit the enhanced surface plasmon resonance of Ag, variously modified, are reported [38,39,40]. An example of a colorimetric sensor array has been reported by Li et al., who realized a smart system based on plasmonic nanocolorants and chemo-responsive organic dyes for the detection of leaf volatile molecules interfaced with a smartphone [41]. Two more examples of colorimetric sensors reported in the literature are based on ferric nitrate as an active compound for MeSA detection [42,43]. Recently, Shi et al. reported the designing of an optical chamber, with a long optical path, for gas non-dispersive infrared gas sensing [44].

Different UV-spectrometric portable devices for gas sensing are reported in the literature [45].

The research gap in Methyl Salicylate (MeSA) detection for agricultural applications lies in the absence of portable, field-deployable optical sensors capable of low-ppb sensitivity without requiring complex sample preparation, laboratory infrastructure or high operative temperatures.

For all these reasons, here, we propose an alternative strategy, based on the exploitation of UV-Vis absorbance for direct MeSA monitoring at room temperature. To this end, we validate the proof of concept of a spectrometric system by adopting a bench deuterium lamp, a 4 L chamber with an optical path of 16 cm, and a 4H-SiC UV photodiode [46]. SiC, in fact, is the best candidate, being a wide-bandgap semiconductor largely adopted for power microelectronics, and due to its unique electronic, optical, and chemical properties, which are also well suited for sensing applications: UV-specific sensitivity (220–380 nm range), enabling direct detection of MeSA’s UV absorption without interference from visible-light fluorescence; miniaturization potential, demonstrated by prior SiC-based portable devices, which supports integration into handheld systems as demonstrated by Sciuto et al. [47]; and chemical inertness and thermal stability, ensuring durability and its application for gas monitoring in harsh environments [48,49]. The proposed SiC-based UV spectrometric system addresses the research gap by also permitting room-temperature operation and avoiding sample preparation.

In preliminary testing, the system achieved 70 ppb sensitivity to MeSA—comparable to stress-induced plant emissions. This performance surpasses that of many existing sensors while avoiding their drift issues. The approach’s simplicity (direct UV absorption measurement) and compatibility with wireless networks make it a practical solution for real-time, distributed MeSA monitoring in precision agriculture. In addition, a preliminary comparative test with another common VOC, Methyl Jasmonate (MeJA), provides us with the ability to exclude potential interferences with our desired analyte signal at the working wavelength.

## 2. Materials and Methods

### 2.1. Reagents

Methyl Salicylate, with a purity grade ≥ 99% GC (yellow liquid, d = 1.174 g/mL, MW = 152.15 g/mol), Methyl Jasmonate with a purity grade of 95% (colorless-to-pale-yellow liquid, d = 1.03 g/mL, MW = 224.30 g/mol), and Ethanol, of analytical grade, were purchased from Sigma Aldrich and used without further purification.

### 2.2. Experimental Lab System Setup

**Figure 1:**
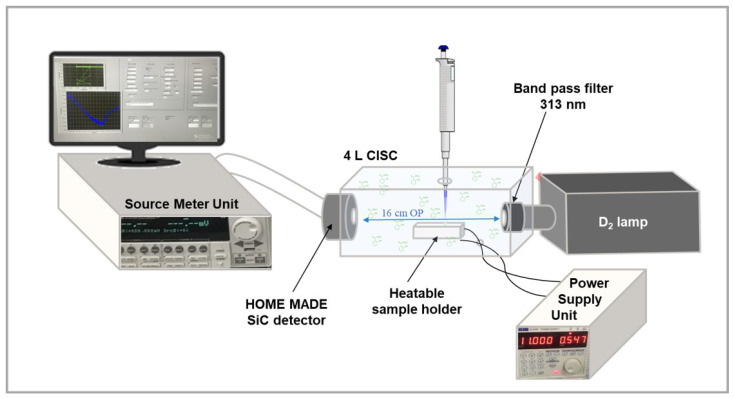
Schematic representation of the experimental setup.

For the laboratory sensing system test, MeSA vapor and MeJA vapor were adopted. Vapor formation was carried out inside a properly modified polypropylene box exploited as a Chemically Interactive System Chamber (CISC) with a 4 L volume and optical path (OP) of 16 cm and using a heatable sample holder. The optical system adopted for the tests consists of a bench deuterium lamp SP-ASBN-D130, equipped with a 10MLF10-313 Mercury Line Bandpass Filter with a FWHM of 11 ± 3 nm, and the homemade low-noise and visible-blind SiC UV detector, which faced each other at the ends of the CISC. A computer remotely connected to Source Meter Unit (SMU) Keithley 2636B (Keithley Instruments, Cleveland, OH, USA) was used to bias the SiC detector (CNR-IMM HQ facilities, Catania, Italy) at a fixed negative voltage and to read a related photocurrent in the range of pA. The intensity current values of the UV photodetector were monitored by using appropriate software developed in LabView. To heat the sample, and thus obtain the MeSA vapor, a holder plate was assembled using an aluminum slab containing a 20 Ohm armored resistor with a power dissipation of 25 W operated by a TTi QL355P power supply unit (Thurlby Thandar Instruments Ltd., Huntingdon, UK). The assembled heatable plate was calibrated using a thermocouple to obtain the curve of temperature versus resistor voltage, not reported for brevity. Some tests were performed without heating the sample. A schematic representation of the entire system is depicted in Figure 1.

### 2.3. Adopted Source and SiC Detector: Electro-Optical Parameters and Properties

**Figure 2:**
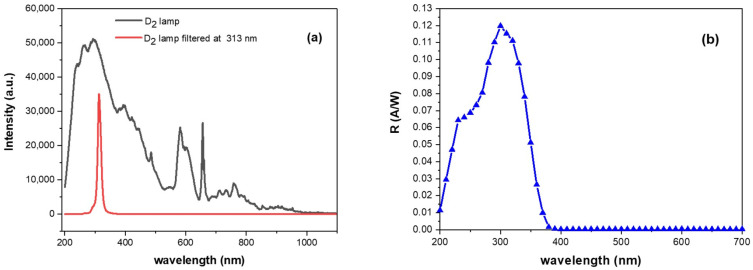
(**a**) Overlapping spectral profiles of deuterium lamp with 313 nm filter (red line) and without (black line). (**b**) Responsivity spectrum of SiC photodetector at 10 V reverse polarization.

Lamp emission spectra were collected with and without a filter and, to appreciate the sharpening of the peak at 313 nm, their overlapping is reported in Figure 2a. Spectra were acquired by using a multimodal UV-vis fiber linked to an AvaSpec-HS2048XL-EVO spectrometer (Avantes B.V., Apeldoorn, The Netherlands). The two reported profiles were recorded with two different integration times of 50 and 10 milliseconds, respectively, due to the different intensity of the transmitted signal trough the bandpass filter. The filtered spectrum exhibits, as expected, a peak at 313 nm with a FWHM of 12 nm. The light signal is revealed by a SiC detector. It was fabricated at CNR-IMM HQ facilities (Catania, Italy) on an n^-^-type 4H-SiC epitaxial layer, about 11 μm thick with a 10^14^ cm^−3^ N dopant concentration, grown by LPE–Italy Silicon Carbide Epi Technologies onto an n-type heavily doped (n + 10^19^ donor/cm^3^ type) 4H-SiC substrate. The thickness and the epi-layer dopant concentration were selected in order to obtain a complete depletion of the epilayer by applying less than 13 Volt. An ohmic contact on the sample back side was formed by sputtering of a 200 nm thick nickel film, followed by a rapid annealing at 1000 °C in a N_2_ ambient. The Schottky contact on the device front side was obtained by sputtering a 100 nm Ni thick film, with an interdigit geometry obtained by combining standard optical lithography and highly selective metal etch. A rapid thermal processing at 700 °C in the N_2_ ambient was used for the treatment of the front Schottky barrier and Ni_2_Si formation [50,51]. The device exhibits a square geometry, with a total area of about 1 cm^2^ and an active area of about 0.72 cm^2^ directly exposed to the impinging radiation.

Detector optical characterization in a continuous wave was performed by using a wide-spectrum and low-flux Xenon lamp (Avantes B.V., Apeldoorn, The Netherlands), a CVI/Digikrom DK240 monochromator (Spectral Product, Albuquerque, NM, USA), a 100 μm diameter core optical fiber (Avantes B.V., Apeldoorn, The Netherlands) with a focusing system and a commercial Ophir-Optronics power meter (Ophir Spiricon Europe GmbH, Darmstadt, Germany). The optical response was investigated as a function of the SiC diode biasing condition and a working reverse polarization of 10 V was identified as the best compromise in terms of low dark current and optical response.

The responsivity spectral profile at 10 V reverse bias, reported in Figure 2b in the range between 200 and 700 nm, was obtained by subtracting the dark current from the photocurrent measured at different wavelengths and referred to the incident optical power. The SiC detector exhibits a response peak of about 0.12 A/W at a 300 nm wavelength and visible blindness, as widely reported in previous work [47].

**Figure 3:**
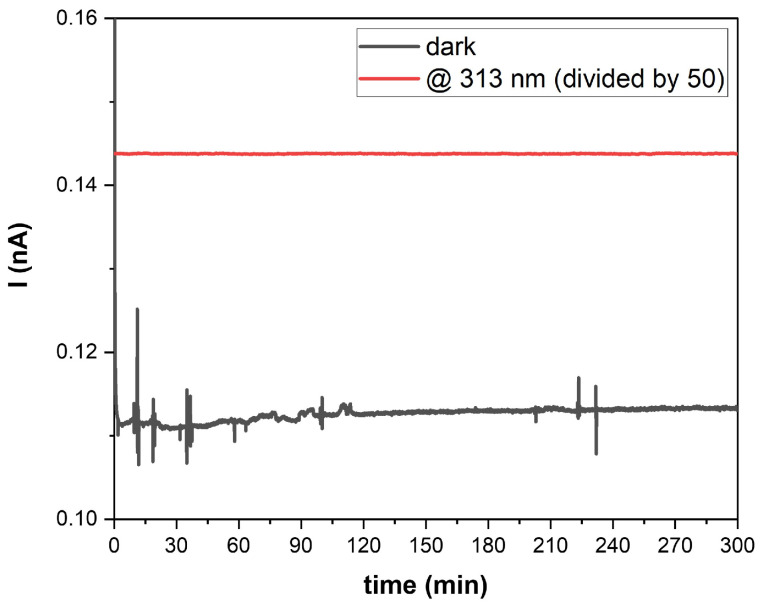
Intensity current signal vs. time overlapping collected by SiC detector in dark (black line) and in 313 nm light (red line) conditions in presence of air in the CISC at RT.

Moreover, a stability test of the entire system was performed both at room temperature and by heating the plate of the sample holder, in the presence of air in the CISC. The trend of the SiC diode photocurrent I was monitored for 300 min, in dark and in UV-light conditions. No appreciable differences in terms of current variations between the two operative temperatures (room temperature and with the heating plate at 72 °C) were registered. For simplicity, in Figure 3, the registered current signals I acquired at room temperature, in dark and in light conditions are reported, which show that the lamp, as well as the detector, are stable for the entire duration of the stability test: a reasonable electrical noise in the range of few pA is observable in the photodetector current due to absence of electrical shielding of the experimental setup.

### 2.4. Procedure and Photo-Current Measurement Conditions

The current intensity reduction of a SiC photodetector in detecting MeSA operates on the principle that the detector’s photocurrent, generated by UV illumination, in this case at 313 nm, decreases when MeSA molecules absorb or interact with the incident UV light. When MeSA is present in the air near the photodetector, it partially absorbs or scatters the UV photons before they reach the active SiC surface, thereby reducing the number of revealed photons. This leads to a measurable decrease in the photocurrent intensity proportional to MeSA concentration, allowing for direct detection without requiring sample preparation. Two different experimental sets of the MeSA samples in air were performed at a controlled temperature of 72 °C and at room temperature. Particularly of note, heating was exploited for a rapid formation of the MeSA vapor for the laboratory sensing test.

The first series was carried out by dispensing, with a micropipette, increasing amounts equal to 0.25, 0.5, 1, 1.4 and 2 μL of liquid MeSA onto the sample plate holder and switching on the heating immediately to heat the liquid analyte and obtain the vapor in the CISC. The photocurrent I was monitored during a long period, starting the measurement before the introduction of the VOC in the chamber. In different test runs, similar values of the starting photocurrent were registered; due to the experimental setup discussed in previous lines and sketched in Figure 1, the starting value of the photocurrent is not the same in all the runs performed. However, as evidenced by stability tests, both dark and photocurrents were stable in each test run. Photocurrent intensity reduction versus time was monitored in each test run, as the substance gradually evaporated, and collected for a minimum of 40 min up to a maximum of 60 min, depending on the signal trend behavior. Each MeSA concentration test was assessed in triplicate and between one measurement and the next, the sample holder was cleaned with ethanol, leaving it to naturally evaporate by opening the chamber, and allowing it to cool to room temperature. The second series was conducted by maintaining the sample holder at room temperature (RT). Then, 10 μL of MeSA were spotted onto the sample plate holder, and in this case, Salicylate evaporates naturally in the CISC. The detection signal was monitored over 270 min. Separately, a test in the same operative conditions was performed on MeJA by spotting 18 μL.

## 3. Results and Discussion

To demonstrate the feasibility of the proposed solid-state-based UV spectrometric system (for a direct detection of MeSA in agriculture), we conducted experimental studies using a custom-designed benchtop optical system. The system integrated a SiC photodetector within a compact optical setup equipped with a deuterium light source and a 313 nm optical filter for selective excitation and detection of MeSA, as illustrated in Figure 1.

As is well established, MeSA exhibits characteristic absorption signals in the ultraviolet–visible (UV-Vis) region [52] due to its molecular structure, primarily arising from conjugated π–electron systems. The UV-Vis absorption spectrum of MeSA typically shows bands at specific wavelengths, corresponding to electronic transitions within the molecule. The most prominent absorption peak occurs in the region 230 ÷ 240 nm, attributed to the π–π* transition of the aromatic ring system. An equally significant but broader absorption band appears in the 280 ÷ 330 nm range, assigned to the n–π* transition. This transition involves the excitation of electrons from non-bonding (n) orbitals to the antibonding (π*) orbitals of the conjugated system. In Methyl Salicylate, this transition is centered near 310 nm, even in the vapor phase [53].

Our study focused on detecting MeSA by targeting the 310 nm absorption band, which aligns with the spectral responsivity of the SiC photodetector in this region (see Figure 2b). This choice was further motivated by the availability of solid-state UV sources and the absence of potential MeSA fluorescence interference at this wavelength, as reported by López-Delgado [53].

In our experiments, we monitored MeSA vapor formation and concentration in controlled conditions, simulating an increasing agri-environmental stress scenario that should correspond to a rising MeSA vapor formation. The system was tested at five predefined concentrations, within a 4 L chamber, as listed in Table 1. Optical signal attenuation due to MeSA vapor formation at 72 °C was recorded over time.

**Figure 4:**
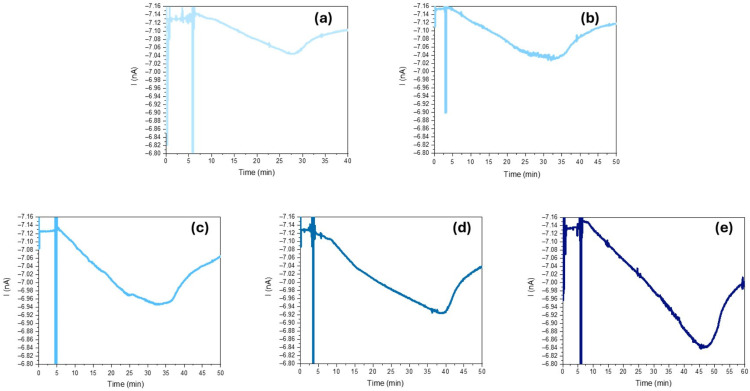
Intensity current trend vs. time observed at increasing MeSA concentration (mol/L): (**a**) 2.94 × 10^−7^ M, (**b**) 9.64 × 10^−7^ M, (**c**) 1.93 × 10^−6^ M, (**d**) 2.71 × 10^−6^ M, and (**e**) 3.86 × 10^−6^ M.

Each concentration test was repeated three times to verify the reproducibility and reliability of the procedure. For simplicity, only one representative curve is shown in the plots (Figure 4a–e) for each concentration, which corresponds to the three trials conducted. Measurements began by recording the detector output signal at room temperature for several minutes to confirm signal stability. After depositing the sample onto the plate, the heater was activated. The full-light photocurrent baseline I_0_, (measured with a 10 V reverse bias applied to the detector) consistently ranged between 7.12 and 7.15 nA (as previously shown in Figure 3).

Upon introducing the sample into the chamber, a distinct signal spike is observed due to the micropipette obstructing the light path during dispensing. This spike appears consistently in all recorded profiles. The temperature of 72 °C was chosen to enhance the analyte’s evaporation rate, ensuring complete evaporation of the deposited compound. As reported by Tevault et al. [54], MeSA volatility (mg/m^3^) increases by approximately 30-fold by passing through 20 °C to 70 °C, enabling time-controlled measurements. As shown in Figure 4a–e, appreciable current variations, ΔI = I_0_ − I, of about 85, 135, 175, 220 and 300 pA were recorded for 2.94 × 10^−7^, 9.64 × 10^−7^, 1.93 × 10^−6^, 2.71 × 10^−6^ and 3.86 × 10^−6^ M, respectively. These results demonstrate a clear correlation between MeSA vapor formation and current reduction ΔI. Optical signal attenuation A is calculated as A = I/I_0_, where I represents the absolute minimum photocurrent recorded during interaction with MeSA vapor and I_0_ is the initial maximum photocurrent (MeSA-free baseline). Optical attenuation values were derived in triplicate for each concentration, with mean values and relative standard deviations summarized in Table 2.

Although the measurements conducted at 72 °C did not replicate realistic agricultural-environmental temperature/kinetic scenarios, they enabled the determination of the apparatus’ sensitivity to the target molecule and demonstrated a clear correlation between MeSA concentration and optical signal attenuation.

**Figure 5:**
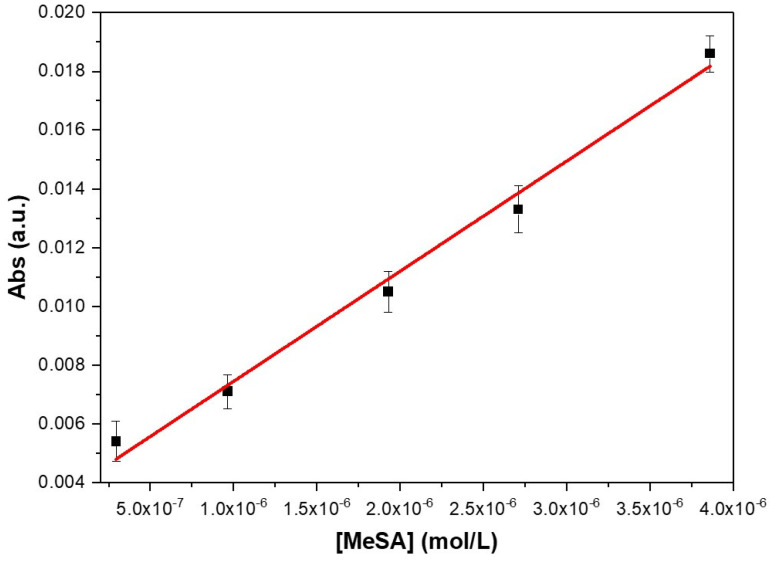
Calibration curve obtained by fitting absorbance vs. MeSA concentration (mol/L).

To establish a calibration curve correlating molar concentration with absorbance, the Lambert–Beer Law was exploited. According to this law, the optical absorbance Abs is directly proportional to the concentration c of the absorbing species, and the optical path lenght d through the sample. Mathematically, this relationship is expressed as follows:

Abs = ε·c·d

where *ε* is the molar extinction coefficient (a wavelength-specific property of the absorbing substance). By converting the measured optical attenuation, A in absorbance and plotting Abs values vs. molar concentration, a calibration curve was generated, as shown in Figure 5.

The linear fit of the data plotted in Figure 5 yielded a R^2^ = 0.992, demonstrating a strong agreement with theoretical predictions. A small non-zero intercept was observed in the calibration curve despite the use of precisely known MeSA concentrations. This deviation is a common occurrence in spectrophotometric methods, and, in our specific case, it could stem from stray light, matrix effects and/or statistical variation inherent to the measurements. 

Using the known optical path length (d = 16 cm, as described Section 2.2), the molar extinction coefficient (ε) at the working wavelength (313 nm), was calculated by the ratio of the slope, of the calibration curve, and d, obtaining ε_(313)_ = 234 ± 15 L·mol^−1^·cm^−1^.

**Figure 6:**
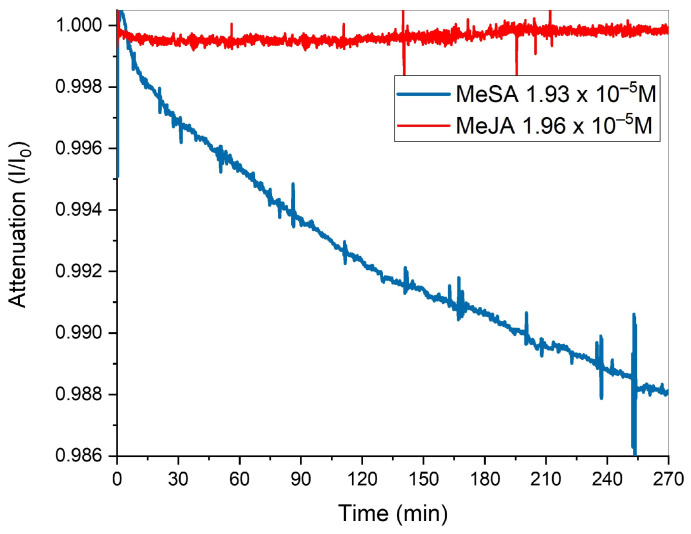
Normalized optical attenuation vs. time overlapping, acquired along MeSA (blue line) and MeJA (red line) vapor formation at RT introduced in the 4 L chamber 10 and 18 μL, respectively.

These results demonstrate successful monitoring of MeSA vapor with a SiC-based UV spectrometric system, paving the way for real-time agricultural emission tracking. A persistent challenge in sensor development is achieving selectivity for the target analyte. The proposed system, currently based on a single wavelength, could be considered weak in terms of false signals due to interfering chemicals. An example of released VOC by plants that might be a potential interferent species is Methyl Jasmonate (MeJA) [5]. MeJA, unlike MeSA, does not exhibit significant optical absorption in this region (at around 310 nm) due to the absence of a conjugated aromatic system; in fact, any weak signals observed in this range are likely due to minor electronic effects from the lactone ring and adjacent functional groups. As demonstrated by Sarang et al., this molecule possesses a negligible *ε* in the absorption wavelength here investigated [55], as well as some Green Leaf Volatiles (GLVs), like *trans*-2-hexen-al, 2-hexen-1-ol and 3-hexen-1-ol [1,2,3,4,5,6,7,8,9,10,11,12,41]. However, minor structural similarities between MeJA and MeSA might still yield partial overlap. To evaluate this, a comparative study at room temperature was conducted. Measurements were performed using 10 µL MeSA and 18 µL of MeJA, ensuring equimolar concentrations in the 4 L chamber, to assess the system’s discrimination capability. The two substances were separately introduced into the chamber and allowed to evaporate naturally at RT. In Table 3, the spotted amount and relative starting concentrations of the two tested analyte molecules are summarized. Relative optical output signals were recorded over a 270 min period. Figure 6 shows the overlapping normalized signals for MeSA and MeJA.

As clearly shown in Figure 6, distinct current reduction trends were observed for the two substances. Despite introducing the same initial molar concentration into the chamber, no appreciable current variation was detected during MeJA evaporation over time. In contrast, a significant current reduction Δ*I* ≈ 90 pA was observed for MeSA, confirming the system’s ability to selectively detect its presence.

The evaporation rate of MeSA under the described conditions (temperature and quantity) is significantly slower compared to previous experiments, where heating accelerated the evaporation kinetics. At room temperature, even with extended monitoring over 270 min, the time was insufficient for complete evaporation of the deposited amounts. Notably, no absolute minimum was observed in the signal, indicating that evaporation was still ongoing. To estimate the quantity of MeSA molecules that transitioned into the vapor phase during this interval, the calibration curve was exploited. A concentration of 4.8 × 10^−7^ M was extrapolated, corresponding to 73 ppb.

At this stage, the assembled optical setup demonstrated a rapid response time and high sensitivity to MeSA, enabling real-time discrimination of MeSA from background noise and from typical VOC interferents such as MeJA.

The development of e-nose and handheld sensors for on-site VOC monitoring with high sensitivity and specificity across multiple application fields in the last few years has received increasing attention [56,57,58,59,60].

**Figure 7:**
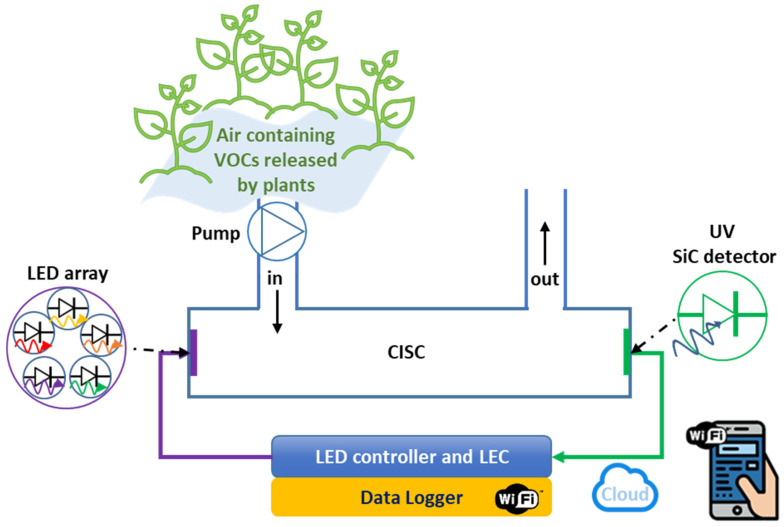
Schematic representation of a potential full solid-state-based portable optical VOC sensor system.

Looking ahead to the development of a portable sensor system for widespread VOC detection in agriculture, a schematic representation is shown in Figure 7. The apparatus should be equipped with a light-emitting diode (LED) or a LED array, a pumping system to convey air and ensure homogeneous distribution within the CISC, a SiC UV detector, and the necessary electronic components for powering, reading, and remote control.

## 4. Conclusions

In conclusion, this work presents the first example of spectrometric measurements for the optical monitoring of MeSA, representing a proof of concept for the exploitation of SiC solid-state technology in the agricultural sector.

The system has demonstrated stability and high sensitivity, detecting MeSA concentrations as low as 2.94 × 10^−7^ M—levels comparable to those emitted by plants under stress—corresponding to tens of ppb. To our knowledge, this sensitivity is on par with or exceeds that of other emerging sensor technologies, which are increasingly being explored as alternatives to traditional ones. Unlike some other optical detection platforms that can be affected by collateral fluorescence phenomena, SiC sensors are “visible blind,” effectively excluding such interference.

A preliminary sensing test also allows us to exclude the potential interference that could come by the concomitance release of MeJA, allowing it to demonstrate selectivity.

The use of solid-state components in the proposed sensing methodology enables compatibility with harsh environments and the implementation of a wireless, real-time system for remote monitoring, thereby facilitating precision agriculture and improving pest management strategies. Furthermore, SiC technology is well suited for the future integration of sensing, reading, and data-processing electronics.

## Figures and Tables

**Table 1:** MeSA amount, in spotted volume, mass, and relative concentration (in mol/L and ppb) calculated in total volume of 4 L, used for the first series measurements performed at 72 °C.

	Spotted Volume (µL)	Mass (mg)	Concentration (mol/L)	Concentration (ppb)
First Measurements Series at 72 °C	0.25	0.29	2.94 × 10^−7^	72.5
	0.5	0.58	9.64 × 10^−7^	145
	1	1.17	1.93 × 10^−6^	292
	1.4	1.65	2.71 × 10^−6^	412
	2	2.35	3.86 × 10^−6^	584

**Table 2:** Optical attenuations measured in triplicate for any tested MeSA concentrations, with relative mean and standard deviation.

Molar Concentration	Optical Attenuation	Mean	Std Dev σ
2.94 × 10^−7^	run 1 0.98796	0.98772	6.5 × 10^−4^
	run 2 0.98822		
	run 3 0.98699		
9.64 × 10^−7^	run 1 0.98523	0.98459	6.3 × 10^−4^
	run 2 0.98398		
	run 3 0.98455		
1.93 × 10^−6^	run 1 0.97475	0.97544	6.7 × 10^−4^
	run 2 0.97609		
	run 3 0.97547		
2.71 × 10^−6^	run 1 0.97103	0.97058	1.9 × 10^−3^
	run 2 0.97226		
	run 3 0.96845		
3.86 × 10^−6^	run 1 0.95836	0.9577	7.0 × 10^−4^
	run 2 0.95789		
	run 3 0.94998		

**Table 3:** MeSA and MeJA amount, in spotted volume and relative concentrations (in mol/L) calculated in a total volume of 4 L, used for the cross-test performed at RT.

Molecule	Spotted Volume (µL)	Concentration (mol/L)
**MeSA** 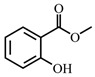	10	1.93 × 10^−5^
**MeJA** 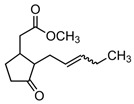	18	1.96 × 10^−5^

## Data Availability

The data are included in the manuscript.

## Abbreviations

The following abbreviations are used in this manuscript:

A	Optical Signal Attenuation (I/I_0_)
Abs	Absorbance
CISC	Chemically Interactive System Chamber
FWHM	Full Width at Half Maximum
GC	Gas Chromatography
GCE	Glassy Carbon Electrode
GLV	Green Leaf Volatile
HPLC	High-Performance Liquid Chromatography
I	Minimum Value light-Photocurrent Intensity
I_0_	Full-Light Photocurrent Intensity
LCR	Inductor Capacitor Resistor
LED	Light Emitting Diode
MeJA	Methyl Jasmonate
MeSA	Methyl Salicylate
MS	Mass Spectrometry
MW	Molecular Weight
MWCNTs	Multiwalled Carbon Nanotubes
NPs	Nanoparticles
QCM	Quartz Crystal Microbalance
rGO-PDADMAC	Reduced Graphene Oxide/Poly (Diallyldimethyl Ammonium Chloride)
RT	Room Temperature
SAR	Systemic Acquired Resistance
SMU	Source Meter Unit
SPME	Solid-Phase Microextraction
TMW	Tobacco Mosaic Virus
UV	Ultraviolet
UV-Vis	Ultraviolet–Visible
VOC	Volatile Organic Compound
ΔI	Current Variation (I_0_ − I)
